# The future of meniscus science: international expert consensus

**DOI:** 10.1186/s40634-021-00345-y

**Published:** 2021-03-31

**Authors:** Nicholas N. DePhillipo, Robert F. LaPrade, Stefano Zaffagnini, Caroline Mouton, Romain Seil, Philippe Beaufils

**Affiliations:** 1 Oslo Sports Trauma Research Center, 4014 Ulleval Stadion, 0806 Oslo, Norway; 2 Twin Cities Orthopedics, Edina, MN USA; 3 Rizzoli Orthopedic Institutes of Bologna, Bologna, Italy; 4 Department of Orthopaedic Surgery, Centre Hospitalier de Luxembourg, Clinique d’Eich, Luxembourg city, Luxembourg; 5 Sports Medicine and Science, Luxembourg Institute of Research in Orthopaedics, Luxembourg city, Luxembourg; 6 Human Motion, Orthopaedics, Sports Medicine and Digital Methods, Luxembourg Institute of Health, Luxembourg city, Luxembourg; 7 Centre Hospitalier de Versailles, Versailles, France

**Keywords:** Meniscus repair, Orthobiologics, Meniscal extrusion, Knee osteotomy, Biomechanics, Posttraumatic osteoarthritis, Orthopedic medical devices

## Abstract

**Purpose:**

The purpose of this study was to evaluate the main focus areas for research and development for furthering the state of meniscus science in 2021.

**Methods:**

An electronic survey including 10 questions was sent in a blind fashion to the faculty members of the *5*^*th*^* International Conference on Meniscus Science and Surgery*. These faculty served as an expert consensus on the future of research and development areas of meniscus science. Survey responses were analyzed using descriptive statistics and ranking weighted averages were calculated to score survey questions.

**Results:**

Of the 82 faculty, 76 (93%) from 18 different countries completed the survey (84% male, 16% female). The highest ranked future research and development focus areas were meniscus repair, biologics, osteotomy procedures, addressing meniscus extrusion, and the development of new therapies for the prevention of posttraumatic osteoarthritis. Currently, the most ‘valuable’ type of biologic reported for meniscus treatment was platelet-rich plasma. The main reported global research limitation was a lack of long-term clinical outcomes data. The most promising emerging medical technologies for improving meniscus science were 3-D printing, personalized medicine, and artificial implants.

**Conclusions:**

This survey suggests that the future of meniscus science should be focused on meniscal preservation techniques through meniscus repair, addressing meniscal extrusion, and the use of orthobiologics. The lack of long-term clinical outcomes was the main reported research limitation globally for meniscus treatment. Future product development utilizing emerging medical technologies suggest the use of 3-D printing for meniscal transplants/scaffolds, personalized treatment, and bioengineering for artificial implants.

**Level of Evidence:**

Level V.

**Supplementary Information:**

The online version contains supplementary material available at 10.1186/s40634-021-00345-y.

## Background

The advancement of meniscus treatment through scientific research can be attributed to the current success of clinical treatment strategies for patients with meniscus-associated pathologies. Understanding the structure and function of the meniscus has played a pivotal role in the evolution of meniscus science [1, 16]. In 1884, Sutton referred to the menisci as ‘functionless’ remnants of intra-articular leg muscles [48]. In 1948, the first degenerative changes of the knee joint following meniscectomy were reported by Fairbank [11]. By 1982, Arnoczky and Warren recognized that the menisci were one of the ‘most important’ structures determining the future of the knee joint [1]. This century-long transition of the conceptualization of the meniscus from being ‘functionless’ to ‘most important’ was through the evolution of scientific research and innovation, particularly involving the discovery of the increased risk of joint degeneration associated with the loss of meniscus tissue [6, 39].

The state of meniscus science has advanced immensely with the evolution of scientific technologies, such as the advent of the arthroscope, which has led to an improved understanding of the meniscus for the longevity of the knee joint [17, 50]. As a result of both technological and research advancements, a paradigm shift from meniscus resection to meniscus repairs was established [2, 36, 45]. The goal of any technological evolution is to meet the needs and expectations of its users—this is the same goal with orthopaedic surgery and the evolution of meniscal treatment [3]. Thus, understanding the key focus areas for future research and development may lead to accelerated technological innovation in the field of meniscus science [10, 26]. Therefore, the purpose of this study was to evaluate the main focus areas for research and development for furthering the state of meniscus science in 2021. We accomplished this by surveying a sample of the top scientific experts from an international consensus on meniscus science.

## Methods

### Survey development

This study was approved by the organizing committee members of the *5*^*th*^* International Conference on Meniscus Science and Surgery* prior to being conducted. An electronic survey including 10 questions was sent in a blind fashion to all of the faculty members of the *5*^*th*^* International Conference on Meniscus Science and Surgery* (Supplement 1)*.* These faculty were primarily orthopedic surgeons. The survey questions were developed by the current authors according to previous trends in the literature regarding meniscus science and also by expert opinionated knowledge from years of clinical practice. A cover letter that accompanied the survey stated the purpose of the survey and ensured anonymity. All contacted participants had the opportunity to decline the survey. The survey was sent out and responses were collected from October 2020 to December 2020.

Therapies for the prevention of posttraumatic osteoarthritis (PTOA) were defined as any intervention designed to avert or avoid OA development in high-risk patient populations. Symptom resolution therapies were defined as any therapy focused on reducing the symptomatology of patients with existing OA, including pain, stiffness, swelling, joint range-of-motion, muscle weakness, fatigue, joint instability, and pain-related psychological distress. Disease-modifying therapies were defined as any treatment that focused on retardation of OA (slowing the speed of progression), a complete halt in disease progression, or a reversal in disease progression (regeneration of targeted tissue).

### Statistical analysis

Data were prospectively collected via an online survey tool (www.surveymonkey.com). They were extracted from the online survey database and summarized. Standard descriptive statistics were performed. Certain focus areas were ranked (low to high) among the respondents from a score of 1 (‘Not Helpful’) to a score of 5 (‘Most Helpful’). Weighted averages were then calculated to provide a statistical datapoint for these questions. For questions that were not weighted, multiple answers were allowed and thus the sum of such questions was not equal to 100%.

## Results

**Fig. 1:**
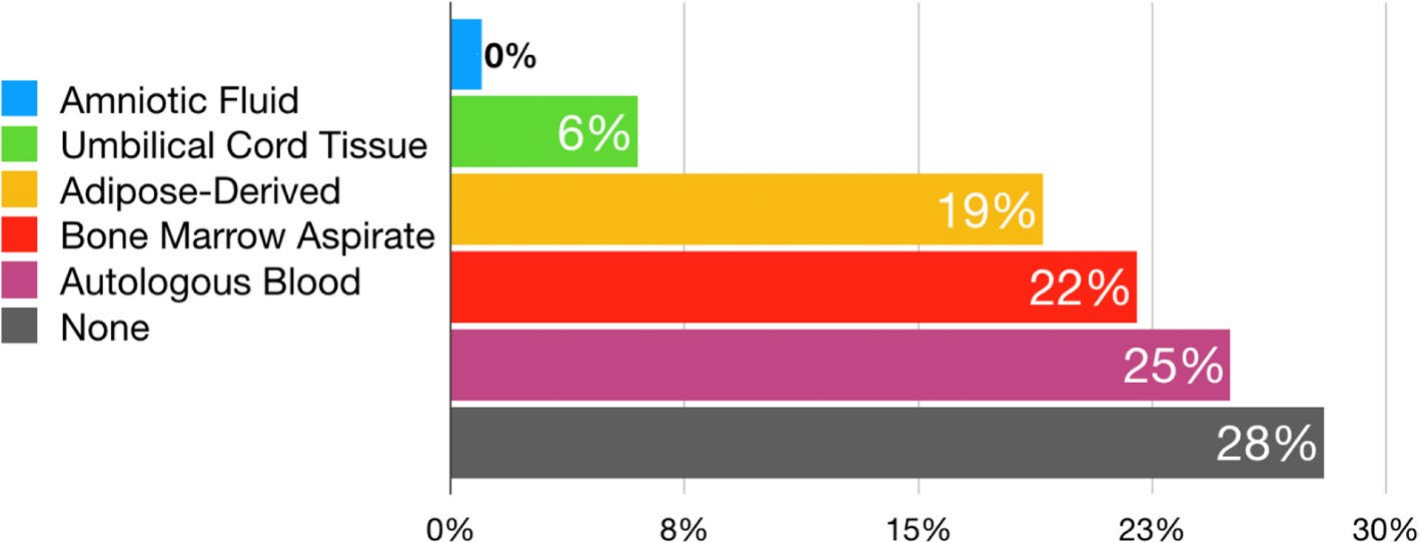
Type of biologics reported as currently ‘most valuable’ for meniscus treatment

Of the 82 faculty members, 76 (93%) from 18 different countries completed the survey (84% male, 16% female). Sixty-four (84%) individuals of the expert panel were orthopaedic surgeons while 12 (16%) were scientists/physiotherapists. The top 3 ranked focus areas of research and development for furthering the state of meniscus science were: meniscus repair (weighted average: 4.55), biologics (weighted average: 4.15), and surgical medical devices (weighted average: 4.00) (Table 1). Studying clinical outcome parameters (64%) was reported as the most ‘important’ focus area for improving clinical outcomes for meniscus repair. The reported most ‘valuable’ biologic for meniscus treatment was autologous blood (25%), including platelet-rich plasma (PRP). The reported least ‘valuable’ biologic for meniscus treatment was amniotic fluid (0%). However, the majority of respondents (28%) reported that none of the current biologics were ‘valuable’ for meniscal treatment (Fig. 1).

**Table 1:** Primary future research and development focus areas with weighted averages^a^

Biologics	4.15
Surgical medical devices	4.00
Meniscus engineering	3.84
Meniscus transplantation	3.63
Nonsurgical medical devices	2.63
Meniscus repair	4.55

^a^Ranking weighted averages: (1) not helpful—(2) low yield—(3) average yield—(4) moderate yield—(5) most helpful

Addressing meniscal extrusion (52%) was reported as the main focus area for improving meniscal allograft transplantation (MAT), followed by improved patient selection (42%) and the utilization of biologics for improved graft incorporation and healing (42%). Osteotomy procedures (weighted average: 4.13), MAT (weighted average: 4.10), and biologics (weighted average: 3.83) were the top 3 ranked focus areas for treating patients with meniscal deficiency. Regarding meniscal preservation, meniscus repair (weighted average: 4.66), intra-articular repair devices (weighted average: 4.16), and biologics (weighted average: 3.97) were reported as the top ranked focus areas. The top ranked focus areas for treating patients with meniscus-associated PTOA were surgery (osteotomy, MAT, cartilage resurfacing; 64%), biologics (55%), pharmaceuticals (27%) and nonsurgical medical devices (unloader bracing and supports; 27%).

**Fig. 2:**
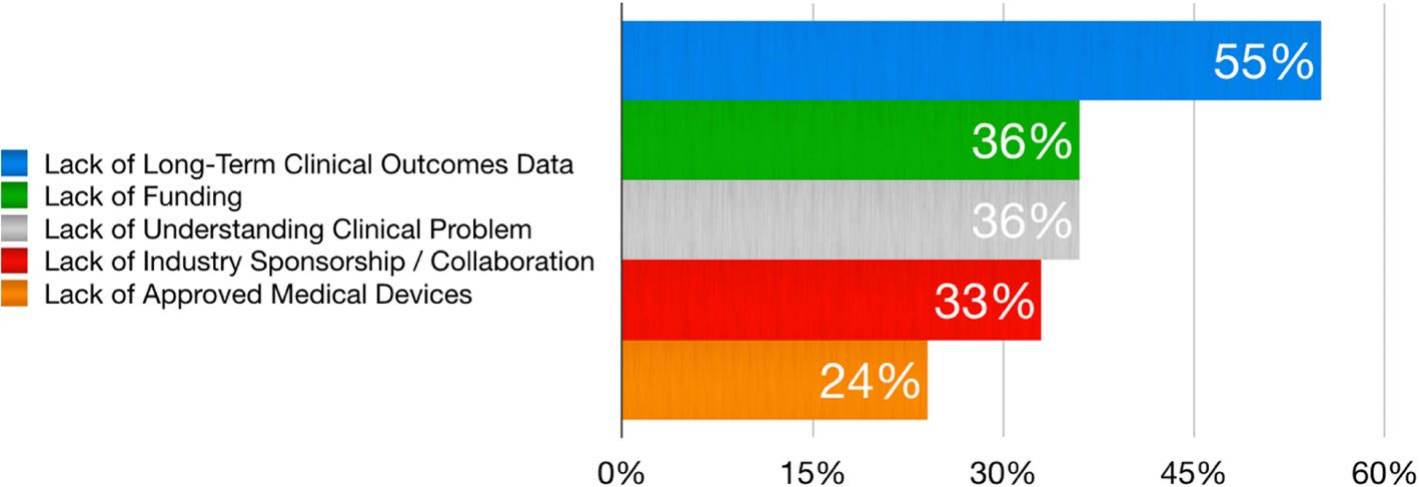
Current global research limitations for improving clinical outcomes in meniscal tear/deficient patient populations

**Fig. 3:**
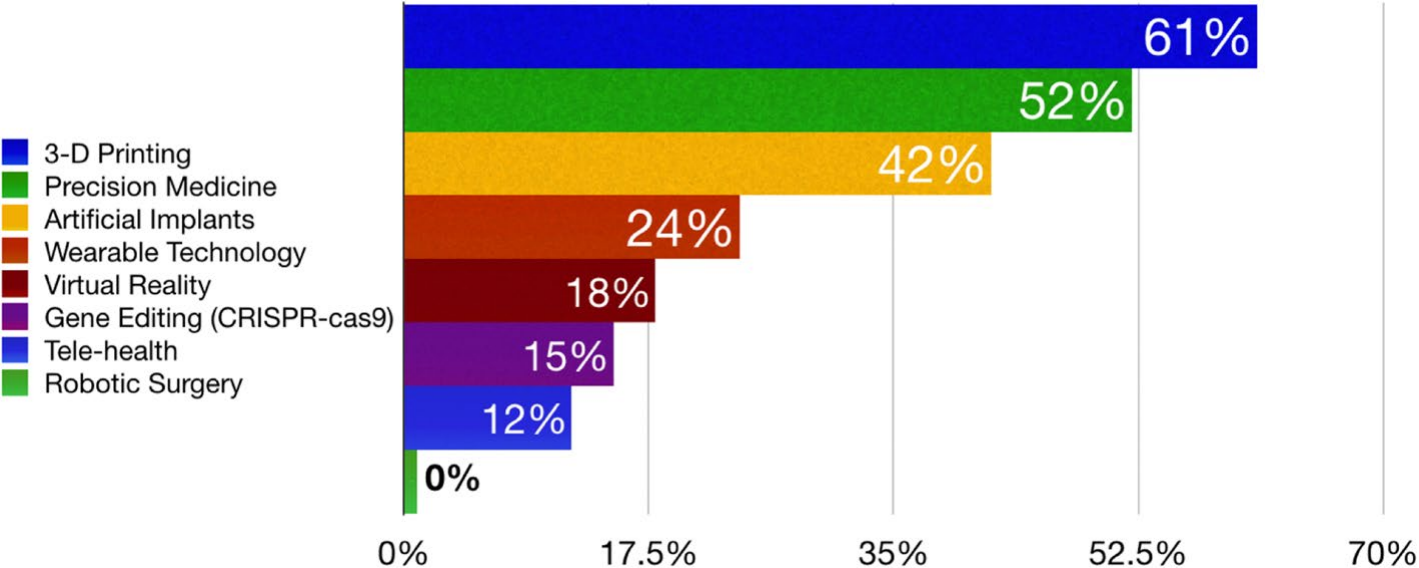
Top ranked emerging medical technologies to have the greatest impact for furthering meniscus science

The main focus area for developing new therapies in treating patients with meniscus-associated PTOA was prevention (64%), followed by the development of disease-modifying drugs (24%) and symptomatic management therapies (12%). The main current global research limitations for improving clinical outcomes in meniscus tear/deficient patient populations were lack of long-term clinical outcomes data (55%), lack of funding (33%), and lack of understanding the clinical problem (33%) (Fig. 2). The use of 3-D printing for meniscal transplants/scaffolds (61%), personalized medicine (52%), and artificial implants (including bioengineering, nanoparticles, and synthetic devices; 43%) were reported as the top ranked emerging medical technologies to have the greatest impact for furthering meniscus science (Fig. 3).

## Discussion

The most important findings of this survey were that the highest ranked future research and development focus areas included meniscus repair, biologics, osteotomy procedures, addressing meniscus extrusion, and the development of new therapies for the prevention of PTOA. Currently, the reported most ‘valuable’ type of biologic for meniscus treatment was PRP, while amniotic fluid was reported as least ‘valuable’. The main reported global research limitation was lack of long-term clinical outcomes data. Finally, the most promising emerging medical technologies for improving meniscus science in the twenty-first century were 3-D printing, personalized medicine, and bioengineering of artificial implants.

This meniscus expert panel collectively suggests focusing future research and development on meniscal preservation through meniscus repair and the use of orthobiologics. Recent evidence including basic science and expert consensus statements have led to an improved understanding of treating meniscal lesions and use of biologic augmentation [4, 14, 20, 26, 28, 36, 40, 45, 47]. Both clinical outcomes data and animal models indicate promising results for meniscal repair and the potential for improved meniscal healing with biologic augmentation [23, 31, 35, 37]. Additionally, this survey suggests that future research and development should focus on intra-articular devices to further improve meniscal repair and enhance the delivery and sustainability of biologics inside the knee joint. Previous research has demonstrated the evolution of meniscal repair strategies, evident by the transition from inside-out techniques to all-inside repair devices [43, 49]. Continual development of biomedical devices involving meniscus repair and biologics delivery are of high importance based on this expert panel survey.

This study demonstrated that biologics, specifically PRP, were consistently ranked as a leading focus area within each domain of meniscus science. This coincides well with current clinical practice apparent by the expanding use and global interest in utilizing biologics for treating an array of musculoskeletal disorders [34, 35, 42, 44, 47]. However, despite the increased interest and consensus for more research and development of biologics in meniscus science, the majority of respondents in this survey reported that biologics are currently not valuable for the treatment of meniscal injuries. This criticism is perhaps due to the unproven regenerative capacity of orthobiologics in clinical studies, despite promising evidence in basic science and animal models [31, 35, 44, 47, 52]. Therefore, the question remains “how” to advance from a technology development perspective. This should involve parallel discovery among clinicians and scientists with collaboration and investment from both the public and private sector.

While new discoveries and sophisticated research methods will undoubtedly continue to contribute to the evolution of orthobiologics, the clinical utility of such therapeutics remain partially limited due to the limited cellular manipulation of biologic products for human use [22, 32, 33]. Therefore, one technological approach for improving the clinical efficacy of biologics in meniscus science is through the development of novel drug delivery platforms. Currently, the major challenges affecting successful delivery of biologics within joints includes rapid clearance of drugs due to passive release and lack of response to the body’s natural physiologic loading mechanisms [38]. Consequently, self-regulating drug delivery systems designed specifically for the mechanical environment of musculoskeletal tissues wherein physiologic feedback actively controls release kinetics have been developed as a solution to overcome this clinical barrier [29]. Controlled drug delivery for musculoskeletal environments show promise in a variety of orthopaedic conditions, including meniscal tears and the consequential degenerative cascade of PTOA [7, 25, 30].

This survey demonstrated high interest in the development of preventative therapies in patients with meniscus-associated PTOA. It is known that there is currently no treatment strategy that can prevent the progression of OA after injury and many treatment options may provide only partial symptomatic relief [41]. The clinical need for improved treatments in patients with meniscus tears and PTOA is clearly evident [12, 18, 20, 23, 24]; meanwhile the technology seeds in bioengineering show great promise for meniscus application in early development phases [13, 15, 21, 27, 29, 51]. Therefore, combining both a clinical need- and technology seed-driven approach may allow for accelerated innovation [8, 9, 19], especially in the domain of meniscus science and knee PTOA. Consequently, future research aimed to optimize biologically-targeted delivery systems may improve the efficacy of current orthobiologics while also assisting in the prevention of knee PTOA. Yet, preserving the meniscus through surgical repair (when indicated) remains the number one priority [12, 18, 23, 40, 45].

Addressing meniscal extrusion was a key focus area for research and development as indicated by this meniscus expert panel. Prior research has shown meniscal extrusion to be a determinant of success for corrective surgery in both meniscal repair and transplantation. Specifically, by addressing meniscal extrusion and successfully relocating or recentering the meniscus in its native anatomic position may best restore the load bearing and force reduction functions of the meniscus. Future research may focus on improving surgical techniques and developing medical technologies that allow for enhanced fixation of the meniscus to address this joint extrusion.

Currently, the main global research limitation in the field of meniscus science is the lack of long-term clinical outcomes as reported by the expert panel. This survey suggests the need for long-term monitoring programs of meniscal tear patients including registry data and multi-center, international collaborations. The future of clinical care is not only dependent on emerging technology, such as 3-D printing and artificial implants, but also relies heavily on interdisciplinary collaboration and innovation [4, 5, 14, 27, 46].

There were limitations of this study inherent to that of a survey. Therefore, the subjective perspectives of the survey respondents cannot be validated with evidence-based recommendations. Furthermore, these practices may be adopted into future research studies for validation. Additionally, there may be inherent bias introduced as a result of an opinionated survey from primarily orthopedic surgeons, thus the results should be interpreted with caution.

## Conclusions

This survey suggests that the future of meniscus science should be focused on meniscal preservation techniques through meniscus repair, addressing meniscal extrusion, and the use of orthobiologics. The lack of long-term clinical outcomes is the main reported research limitation globally for meniscus treatment. Future product development utilizing emerging medical technologies suggest the use of 3-D printing for meniscal transplants/scaffolds, personalized treatment, and bioengineering for artificial implants.

## Supplementary Information

**Additional file 1** Supplement 1 The survey questionnaire with responses from expert meniscus consensus*.

## Data Availability

All data generated or analyzed in this study are included in this published article.

## Abbreviations

PTOA: Posttraumatic osteoarthritis
PRP: Platelet-rich plasma
BMC: Bone marrow concentrate
MAT: Meniscus allograft transplant
