# Supplementary material for: The future of meniscus science: international expert consensus
Source: J Exp Orthop. 2021 Mar 31;8:24. doi: 10.1186/s40634-021-00345-y (PMC8012449; doi:10.1186/s40634-021-00345-y)
Supplement: Supplementary file 1 — Additional file 1 Supplement 1 The survey questionnaire with responses from expert meniscus consensus*. [file 40634_2021_345_MOESM1_ESM.docx]

**Supplement 1 The survey questionnaire with responses from expert meniscus consensus***

| **Q1: In order to improve clinical outcomes for patients undergoing meniscal repair, what research areas should we focus on?**   1. Basic science - anatomy (e.g. quantitative measurements, histology, vascularization) **(42.4%)** 2. Basic science - biomechanics (e.g. repair techniques, tear consequences, effect of meniscal extrusion) **(58.0%)** 3. Clinical science - diagnosis (e.g. preoperative and intraoperative) **(21.2%)** 4. Clinical science - addressing outcome parameters (e.g. rehabilitation, meniscal extrusion, patient characteristics, return to sport) **(64.0%)** 5. Clinical science - large data healthcare system analysis (e.g. economics, cost-effectiveness, regulatory issues) **(33.3%)** |
| --- |
| **Q2:** **In order to further the current state of meniscus science, please rate the following focus areas of future research and development:**   1. Biologics = **4.15** 2. Meniscus repair = **4.55** 3. Meniscus transplantation = **3.63** 4. Meniscus engineering = **3.84** 5. Nonsurgical medical devices = **2.63** 6. Surgical medical devices = **4.00** |
| **Q3:** **In your opinion, what type of cellular biologic is most valuable for meniscal treatment (repair, regeneration, or preservation)?**   1. Adipose-derived stem cells **(18.7%)** 2. Autologous blood (PRP) **(25.0%)** 3. Bone marrow aspirate concentrate **(22.0%)** 4. Amniotic fluid **(0.0%)** 5. Umbilical cord tissue **(6.2%)** 6. None **(28.1%)** |
| **Q4: What focus areas need to be targeted in research for improving clinical outcomes in meniscal transplantation surgery? In answering this, please think what factors 'most' affect meniscal transplant graft survival.**   1. Addressing meniscal extrusion **(52.0%)** 2. Concomitant osteotomy procedures **(33.3%)** 3. Improved patient selection **(42.4%)** 4. Concomitant cartilage procedures **(39.4%)** 5. Use of synthetic grafts **(6.1%)** 6. Improved transplantation techniques **(27.3%)** 7. Utilization of biologics for improved graft incorporation and healing **(42.4%)** 8. Changing postoperative physical therapy protocols **(12.1%)** |
| **Q5: In regard to treating meniscal deficiency, please rate the following focus areas for future research and development:**   1. Meniscus allograft transplant = **4.10** 2. Osteotomy procedures = **4.13** 3. Collagen scaffolds = **3.45** 4. Synthetic meniscal grafts = **3.33** 5. Biologics = **3.83** 6. Physical therapy = **3.00** 7. Nonoperative medical devices (e.g. unloader bracing and supports) = **3.91** |
| **Q6: In regard to meniscal preservation, please rate the following focus areas for future research and development:**   1. Meniscal repair = **4.66** 2. Biologics = **3.97** 3. Osteotomy procedures = **3.55** 4. Cartilage procedures = **3.34** 5. Physical therapy = **2.86** 6. Intra-articular repair devices = **4.16** 7. Intra-articular biologics delivery devices = **3.91** |
| **Q7: In regard to the treatment of posttraumatic osteoarthritis (PTOA) in meniscal tear/deficient patients, which of the following focus areas are most important for research and development:**   1. Pharmaceuticals **(27.3%)** 2. Biologics **(55.0%)** 3. Nonsurgical medical devices (e.g. unloader bracing, supports) **(27.3%)** 4. Arthroscopy **(21.2%)** 5. Arthroplasty **(12.1%)** 6. Surgery (e.g. osteotomy, meniscus allograft, cartilage resurfacing) **(64.0%)** |
| **Q8: In your opinion, as an industry, where should we best focus our efforts in developing new therapies in patients with meniscus-associated PTOA?**   1. Therapies for symptomatic management of existing PTOA **(12.1%)** 2. Therapies for the prevention of future development of PTOA **(64.0%)** 3. Therapies for disease-modifying/reversal of existing PTOA **(24.2%)** |
| **Q9: Please select the following areas of current global research limitations for improving clinical outcomes in meniscal tear/deficient patient populations:**   1. Lack of funding **(36.4%)** 2. Lack of industry sponsorship/collaboration **(33.3%)** 3. Lack of understanding clinical problem **(36.4%)** 4. Lack of long-term clinical outcomes data **(55.0%)** 5. Lack of approved medical devices **(24.2%)** |
| **Q10: Of the top ranked new medical technologies in 2020, please select which technologies may have the greatest impact for furthering meniscus science (select all that apply):**   1. CRISPR-cas 9 (e.g. gene editing) **(15.1%)** 2. Tele-health (e.g. improving accessibility of patient care) **(12.1%)** 3. Virtual reality (e.g. surgical technique practice and education) **(18.2%)** 4. Precision medicine (e.g. personalized treatment) **(52.0%)** 5. Wearable technology (e.g. injury risk, bracing, supports, postoperative monitoring, return to play assessments) **(24.2%)** 6. Artificial implants (e.g. bioengineering, nanoparticles) **(42.4%)** 7. 3-D printing (e.g. meniscal transplants, scaffolds) **(61.0%)** 8. Robotic surgery **(0.0%)** |

*Ranking weighted averages: (1) not helpful - (2) low yield - (3) average yield - (4) moderate yield - (5) most helpful
